# Essential Role of Enzymatic Activity in the Leishmanicidal Mechanism of the Eosinophil Cationic Protein (RNase 3)

**DOI:** 10.1021/acsinfecdis.1c00537

**Published:** 2022-06-22

**Authors:** María Ángeles Abengózar, María Fernández-Reyes, Vivian A. Salazar, Marc Torrent, Beatriz G. de la Torre, David Andreu, Ester Boix, Luis Rivas

**Affiliations:** † Department of Structural and Chemical Biology, Consejo Superior de Investigaciones Científicas (CSIC), Centro de Investigaciones Biológicas Margarita Salas, Ramiro de Maeztu 9, 28040 Madrid, Spain; ‡ Department of Biochemistry and Molecular Biology, Faculty of Biosciences, Universitat Autònoma de Barcelona, 08193 Cerdanyola del Vallès, Spain; § Department of Biomedical Engineering, Universidad de los Andes, Cra. 1E No. 19a-40, Bogota, Colorado 111711, Colombia; ∥ Department of Medicine and Life Sciences, Universitat Pompeu Fabra, Barcelona Biomedical Research Park, Dr. Aiguader 88, 08003 Barcelona, Spain

**Keywords:** membrane disruption, cell-penetrating enzyme, antimicrobial peptide, RNase, protozoa

## Abstract

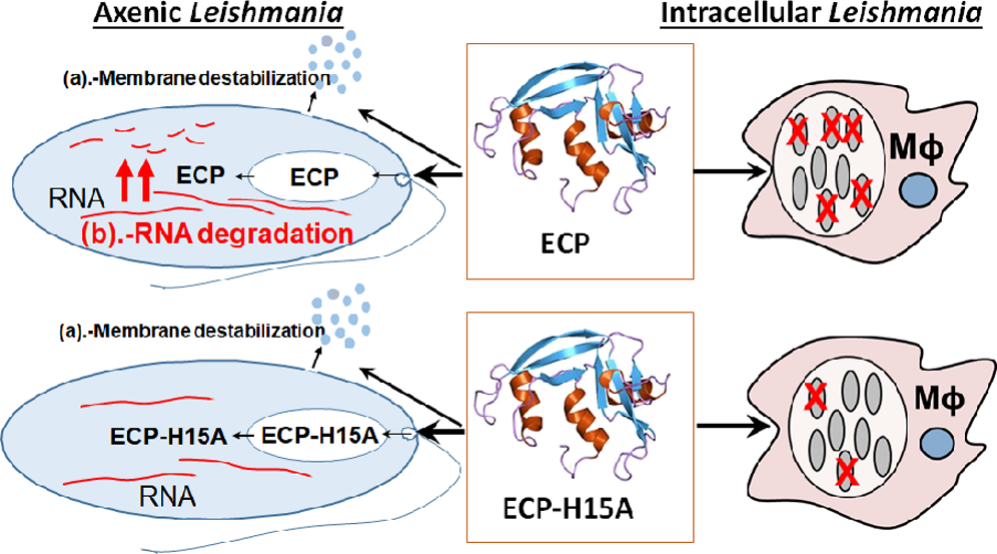

The recruitment of eosinophils into *Leishmania* lesions is frequently associated with a favorable evolution. A feasible effector for this process is eosinophil cationic protein (ECP, RNase 3), one of the main human eosinophil granule proteins, endowed with a broad spectrum of antimicrobial activity, including parasites. ECP was active on *Leishmania* promastigotes and axenic amastigotes (LC_50_’s = 3 and 16 μM, respectively) but, in contrast to the irreversible membrane damage caused on bacteria and reproduced by its *N*-terminal peptides, it only induced a mild and transient plasma membrane destabilization on *Leishmania donovani* promastigotes. To assess the contribution of RNase activity to the overall leishmanicidal activity of ECP, parasites were challenged in parallel with a single-mutant version, ECP-H15A, devoid of RNase activity, that fully preserves the conformation and liposome permeabilization ability. ECP-H15A showed a similar uptake to ECP on promastigotes, but with higher LC_50_’s (>25 μM) for both parasite stages. ECP-treated promastigotes showed a degraded RNA pattern, absent in ECP-H15A-treated samples. Moreover ECP, but not ECP-H15A, reduced more than 2-fold the parasite burden of infected macrophages. Altogether, our results suggest that ECP enters the *Leishmania* cytoplasm by an endocytic pathway, ultimately leading to RNA degradation as a key contribution to the leishmanicidal mechanism. Thus, ECP combines both membrane destabilization and enzymatic activities to effect parasite killing. Taken together, our data highlight the microbicidal versatility of ECP as an innate immunity component and support the development of cell-penetrating RNases as putative leishmanicidal agents.

Leishmaniasis is one of the most important human protozoan diseases with ca. 1,5000,000 new cases per year, and an associated mortality of 7000–15,000 deaths per year.^1^ Its complex pathology is grouped under three major clinical forms, namely, visceral, mucocutaneous, and cutaneous leishmaniasis. This broad clinical diversity is due to both the distinct infecting species of the genus *Leishmania* and the immunological response of the patient.^2^

The life cycle of *Leishmania* is digenetic. It encompasses two major morphological forms, the flagellated promastigote, dwelling inside the gut of its sandfly vector, and the aflagellated amastigote, which lives and replicates inside a parasitophorus vacuole of mononuclear phagocytes. To ensure survival throughout these two harsh yet rather different environments, both major forms differ on their respective cell membrane components, antigenic repertoire and metabolism.^3^

Knowledge about the diverse immune cells involved in host response against *Leishmania* infection has accrued from an early, still highly relevant view^4^ of cross-talk between Th1 and Th2 CD4^+^ subsets with the macrophage to the realization that *Leishmania-*triggered responses encompass not only new CD4^+^ subtypes but also the recruitment of CD8^+^, natural killer cells, basophils, neutrophils, and eosinophils,^5−7^ each with different roles depending on the time and clinical form of leishmaniasis.

In the histopathology of *Leishmania* lesions, recruitment of an eosinophil population near to the infected macrophages, although controversial,^8^ is generally associated with a beneficial influence on the outcome of the infection.^5,9−11^

Eosinophils use their own proteins as effector molecules for their various immune roles^12^ The effect is not confined to the eosinophil, since after eosinophil activation granule proteins can be released into the extracellular medium and acquired by nearby cells, propagating biological activity beyond eosinophils.^13^ Eosinophil cationic protein (ECP, RNase 3), together with major basic protein 1 (MBP-1), and eosinophil-derived neurotoxin (EDN) are the most abundant of such granule proteins, all strongly cationic, and with RNase activity for both ECP and EDN.^12^

Over the last decades, RNase research has expanded from the conventional view of extra-and intracellular RNA control tools to that of regulators of the immune response, hence a key tool in the antimicrobial armamentarium of vertebrates (reviewed in ref (13)). In addition, cationic RNases act not only by intracellularly degrading RNA but also through direct membrane destabilization, depending on the targeted organisms.^14^

Members of the RNase A family are probably the best documented RNases involved in the defense against pathogens.^13^ In terms of antimicrobial role, RNase 3 (eosinophil cationic protein, ECP) is the best characterized. ECP is a small, highly cationic protein (pI = 10.8) with moderate RNase activity compared to other family members such as EDN. ECP is active against a wide range of organisms, from RNA virus, bacteria—including intracellular mycobacteria—to pluricellular helminths.^15−17^ The microbicidal mechanism of ECP against eukaryotic pathogens is puzzling; although RNase activity is essential for antifungal effect on *Candida*,^14^ membrane disruption is involved in antitrypanosomatid activity, as inferred from the scarce inhibition achieved by Rnasin, a recombinant inhibitor of *Trypanosoma cruzi* trypomastigote killing by ECP.^18^ A membrane targeting role was also supported by the lack of correlation between RNase and leishmanicidal activities on *Leishmania donovani* promastigotes in a series of ECP mutant versions harboring single and double replacements for basic residues not involved in RNA hydrolysis.^19^

Under the above criteria, ECP should be viewed as an eukaryotic antimicrobial peptide (AMP). This class of peptides specifically recognizes the target organism mostly on the basis of surface electric charge. The anionic membrane of prokaryotes or lower eukaryotes drives massive binding of cationic AMPs that ultimately kill the cell by disruption of its phospholipid bilayer, whereas higher eukaryotes with zwitterionic plasma membranes are mostly spared.^20^ Indeed, the polycationic character of structural proteins and enzymes recognizing polyanionic nucleic acid scaffolds makes them rather suitable as AMPs. This view is supported by, e.g., the killing of *Leishmania* promastigotes by human histones H2A and H2B, through a membrane disruption process.^21^

The above data are susceptible of two non mutually excluding interpretations. The variation in the number of basic residues in ECP—or its binding to the RNase inhibitor, an acidic protein 3× the size of ECP—may alter membrane binding and further access to parasite cytoplasm, hence precluding a contribution of RNase enzymatic activity to the lethal effect.

Faced with this dilemma, we have opted for a twofold approach. On the one hand, we have evaluated *Leishmania* membrane disruption by ECP and a set of peptide analogues endowed with bactericidal activity, hence an RNase-independent mechanism.^22^ On the other hand, we have compared the leishmanicidal profiles of ECP and its H15A mutant, devoid of RNase activity but preserving the structure of native ECP, in particular the topology of exposed basic residues.^14^

Our results indicate a predominant role of RNase activity in ECP leishmanicidal action. Although membrane-active ECP peptides were leishmanicidal, their specificity was lower than that of the native enzyme. Hence, a role for eosinophils in leishmaniasis (a scarcely researched area, reviewed in ref (5)) is predicated by our data, suggesting RNA degradation—already under study for oncological and other therapies^17,23^—as a feasible target for leishmaniasis treatments.

## Results

### Leishmanicidal Activity of ECP and Related Peptides

Recombinant ECP, plus four ECP *N*-terminal peptides (Table 1), were evaluated as leishmanicidal agents by inhibition of MTT reduction, assayed either upon incubation (IC_50_) or after proliferation of surviving parasites (LC_50_) in the absence of agent, to assess, respectively, short- or long-time cytotoxicity. ECP and the peptides displayed diverse activities against *Leishmania*. Thus, ECP was significantly more potent than ECP(1–45), the peptide replicating the *N*-terminal domain, in contrast to their equipotency in bacteria.^22^ Interestingly, ECP(8–45), a 7-residue shorter version of the *N*-terminal domain, recovered full activity against promastigotes, even with some slight advantage over native ECP (IC_50_ and LC_50_ 3.2 and 3.4 μM, respectively). In contrast, ECP(6–17)-Ahx-(23–36), defined as the pharmacophore for bactericidal activity,^22^ showed a moderate loss of leishmanicidal activity, with viability and proliferation, respectively, dropping to 63.3 ± 2.3 and 61.6 ± 3.0% at the highest concentration assayed (30 μM), precluding calculation of IC_50_ and LC_50_ values within a reliable confidence interval. The similar IC_50_ and LC_50_ values obtained for the peptides suggest that damage to the parasite upon the first 4 h incubation is irreversible.

**Table 1:** Leishmanicidal Activity of ECP and Surrogate Peptides

		promastigote (μM)/[% inhibition @30 μM]^e^		amastigote (μM)/[% inhibition @30 μM]^e^		hemolysis (μM)
reagent	sequence	IC_50_^d^	LC_50_^d^	IC_50_^d^	LC_50_^d^	HC_50_^a^
ECP	a	3.4 (±0.1)	3.4 (±0.2)	17.3 (±1.1)	16.3 (±0.8)	>25
ECP(1–45)	RPPQFTRAQWFAIQHISLNPPRSTIAMRAINNYRWRSKNQNTFLR	8.4 (±3.6)	10.7 (±3.7)	19.1 (±6.8)	>30.0/[23.8±9.8]	11.7 ± 0.2
ECP(8–45)	AQWFAIQHISLNPPRSTIAMRAINNYRWRSKNQNTFLR	3.2 (±0.7)	3.4 (±0.5)	7.5 (±2.3)	>30.0/[49.5 ± 1.9]	10.5 ± 0.2
ECP(24–45)	TIAMRAINNYRWRSKNQNTFLR	8.3 (±1.4)	8.3 (±1.1)	24.4 (±4.9)	>30.0/[25. 8± 1.2]	18.7 ± 0.1
ECP 17Ahx^b^^,^^c^	TRAQWFAIQHIS-Ahx-STIAMRAINNYRWR^b^	>30/[36.3 ± 7.0]	>30/[38.4 ± 8.3]	19.0 (±5.1)	>30.0/[26.1 ± 2.4]	7.3 ± 0.1

a Full amino acid sequence for ECP is available at UniProtKB Database (P12724).

b ECP17Ahx: ECP(6–17)-Ahx-(23–36).

c Ahx—6-aminohexanoic acid.

d IC_50_—half-maximal inhibitory concentration, LC_50_—half-maximal lethal concentration, HC_50_—half-maximal hemolytic concentration.

e For a relative comparison of activities among peptides.

Amastigotes are responsible for the pathological effects of *Leishmania* on vertebrates. To get an unbiased appraisal of the action of ECP and its analogue peptides on amastigotes, without interference of the macrophage as host cell, an *Leishmania pifanoi* axenic amastigote cell line was used. The choice relies on solidly proven similarities between these axenic amastigotes and those from lesions, in terms of morphology, infectivity, metabolism and antigenicity,^24^ and reproducibility throughout time, in comparison with axenic amastigotes obtained from promastigotes by heat and pH stress. Interestingly, the *L. pifanoi* axenic amastigotes were more resistant to ECP (IC_50_ and LC_50_: 17.3 ± 1.1 and 16.3 ± 0.8 μM, respectively) than promastigotes (Table 1). This interstage difference is even higher for the peptides. In fact, the low inhibition obtained for some of the peptides prevented statistical calculation of reliable IC_50_ and LC_50_’s. Likewise, parasite survival values showed a significant reduction for both ECP and the peptides.

Previous work ruled out the toxicity of ECP and the peptides on erythrocytes (hemolysis assay) and on a variety of cell lines: RAW 264.7, THP1-derived macrophages, hepG2 hepatocarcinoma and MRC5 fibroblasts, with IC_50_ values > 50 μM,^16,25^ well above microbicidal concentrations (Table 1). Inhibition of MTT reduction by peritoneal macrophages was 6.1 ± 2.7% for ECP at 30 μM. At this concentration, the highest tested in the study, entry of the vital dye SYTOX Green into macrophages was barely 2% of the value obtained on cells fully permeabilized with 0.1% Triton X-100 (data not included).

A more physiological insight into the leishmanicidal mechanism was gained by treatment of *L. pifanoi* amastigote-infected murine macrophages with ECP labeled with Alexa 488 (ECP-Alexa) 24 h after infection. As shown in Supporting Figure 1, ECP-Alexa is spotted within the parasitophorous vacuoles. Furthermore, the amastigote:macrophage ratio of 3.06 ± 0.28 in control macrophages decreases to 0.67 ± 0.13 in the ECP-treated group (*p* < 0.01, Student’s *t*-test).

### Plasma Membrane Permeabilization of *L. donovani* Promastigotes by ECP and Related Peptides

**Figure 1:**
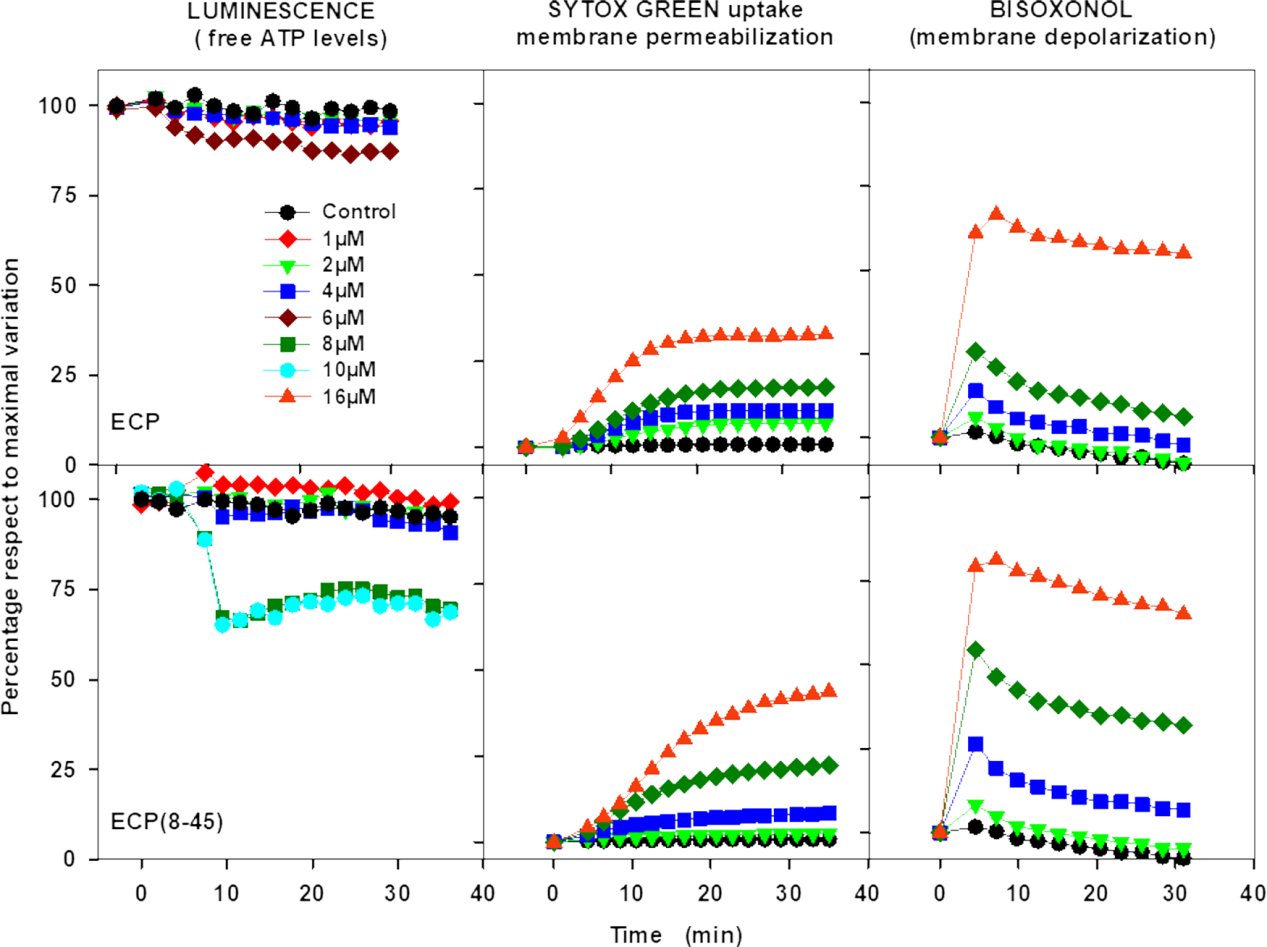
Assessment of plasma membrane permeabilization of *L. donovani* promastigotes by ECP and ECP(8–45); top row: ECP; bottom row: ECP(8–45). Reagents were added at *t* = 0. Variation of the respective parameter is shown on the *y* axes. Luminescence at *t* = 0 was taken as 100%, whereas the negative control consisted in parasites incubated in the absence of luciferase substrate. For SYTOX Green and bisoxonol, 100% fluorescence was the value for parasites treated with 0.1% Triton X-100 or with the AMP CA(1–8)M(1–18) at 3 μM, respectively.

Membrane permeabilization by ECP and ECP(8–45), the analogue with the highest leishmanicidal activity, was assessed by three complementary techniques:

(i) In vivo luminescence of 3-Luc promastigotes^26^ decreased by 40% when incubated with ECP(8–45) at 8 μM, a concentration close to its IC_50_, whereas ECP, even at higher concentration, had no effect, pointing to strong differences in the mechanism of action between the enzyme and its surrogate peptide (Figure 1, left). This suggests that the peptide acts by either direct inhibition of ATP synthesis or by causing a destabilization of the plasma membrane, whereupon ATP would leak into the external medium and/or be used up by ionic pumps striving to restore collapsed ionic gradients.
(ii) To discern which of the above possibilities was correct, entrance of SYTOX Green into the promastigote was measured. At the respective IC_50_’s, ECP(8–45) produced a higher effect than ECP (Figure 1, middle), in agreement with luminescence results. A notable increase in fluorescence was observed within the first minutes after peptide addition, reaching half of the maximum level around 10 min.
(iii) To detect subtler membrane lesions, plasma membrane depolarization was measured by the bisoxonol assay. Maintenance of plasma membrane potentials depends exclusively on ionic gradients across the membrane. As expected, depolarization was higher for ECP(8–45) than for ECP (Figure 1, right). Except for the highest concentrations, depolarization was partially reversible with time, suggesting that membrane damage is only transitory. This effect cannot be monitored by SYTOX Green entry, due to its almost irreversible binding to intracellular nucleic acids. The slower kinetics of SYTOX Green intracellular accumulation requires a higher membrane destabilization than depolarization, which depends on the collapse of ionic gradients across the membrane.

### ECP and ECP-H15A Compared for Leishmanicidal Activity

**Figure 2:**
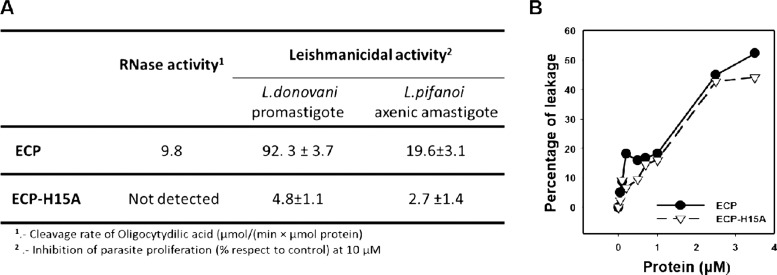
Comparison of ECP and ECP-H15A functionalities. (A) RNase activity was assayed on oligocytidylic acid at 25 °C and expressed as μmol/min per μmol of protein. Leishmanicidal activity was expressed as the percentage of inhibition of proliferation of *L. donovani* promastigotes relative to control at 10 μM protein. (B) Leakage of DOPC:DOPG (3:2 molar ratio) liposomes caused by addition of up to 4 μM ECP and ECP-H15A.

As the above results strongly suggested a leishmanicidal mechanism for ECP other than strict membrane permeabilization, RNase activity was investigated. To this end, the single-mutant ECP-H15A, with essential catalytic His15 replaced by Ala, was assayed. While devoid of RNase activity,^27^ ECP-H15A retains an overall three-dimensional structure (PDB ID: 4OWZ) identical to wild-type ECP, as well as its membrane depolarization and permeabilizing activities.^14^ We corroborated the lack of RNase activity of ECP-H15A, and that its liposome leakage ability was identical to ECP (Figure 2). In contrast, ECP-H15A displayed only residual leishmanicidal activity at a concentration well above the IC_50_ for ECP (Figure 2). At the maximal concentration assayed (30 μM), the inhibition of parasite proliferation by ECP-H15A was 29.7 ± 4.8% and 10.2 ± 2.2%, relative to untreated parasites, for *L. donovani* promastigotes and *L. pifanoi* axenic amastigotes, respectively.

**Figure 3:**
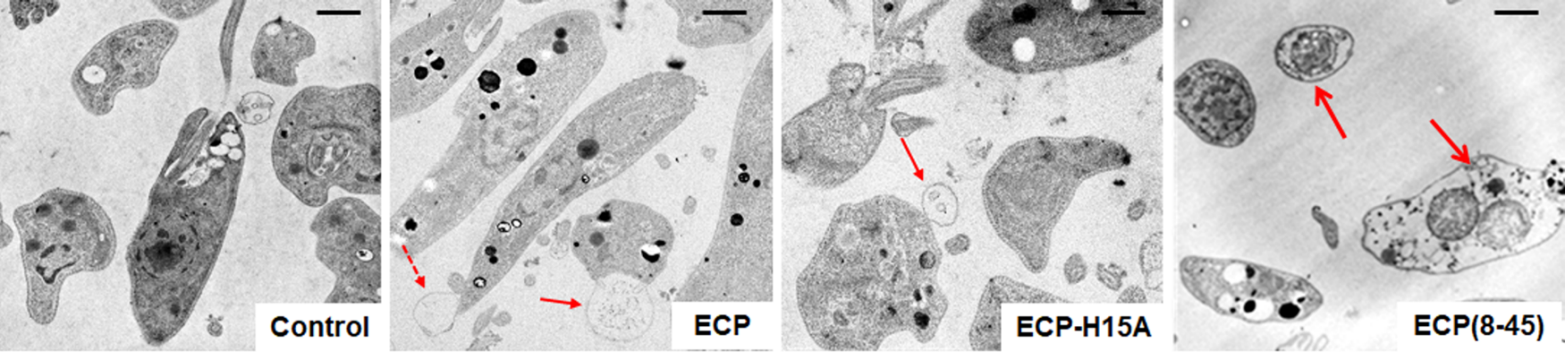
Transmission electron microscopy of *L. donovani* promastigotes treated with ECP, ECP-H15A, or ECP(8–45). Parasites were treated with 4 μM reagent for 4 h in defined medium (HBBS-Glc). Representative images are selected. Arrows point to lesions induced in parasites. Magnification bar = 1 μm.

Next, we visualized the action of ECP and related peptides by electron microscopy, which provides insights into their effect on the morphology and internal structure of the parasite. Damage by ECP and ECP(8–45) was assessed at 4 μM, a concentration near IC_50_. For comparison, ECP-H15A was also assayed at this concentration. After 4 h, parasites incubated with ECP(8–45) showed an all-or-none pattern of membrane disruption (Figure 3), where a population of promastigotes without defined internal organelles and highly translucent cytoplasmic material coexisted with others with apparently unscathed intracellular morphology, whereas others showed membrane bubbles, a typical feature of plasma membrane damage. At this concentration and incubation time, damaged promastigotes also appeared in ECP and ECP-H15A, but the extent and frequency of the morphological damage was much lower, especially for ECP-H15A. Thus, while visible cell damage was roughly estimated for ECP(8–45) at 43 ± 8.0%, for ECP and ECP-H15A, it dropped to 27 ± 3.0 and 6.5 ± 3.5%, respectively, over 200 cells counted in four fields chosen randomly (*p* < 0.01).

### ECP, but Not ECP-H15A, Degrades RNA in *L. donovani* Promastigotes

**Figure 4:**
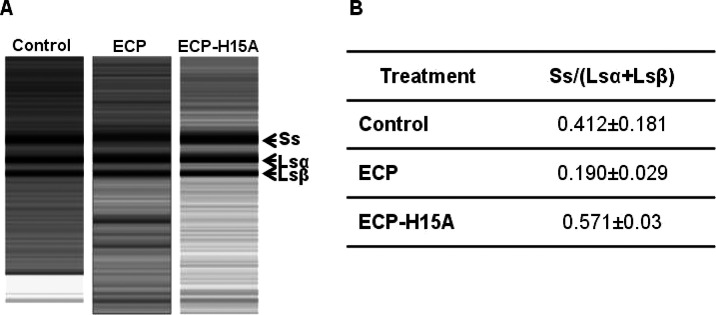
RNA pattern of *L. donovani* promastigotes treated with ECP and ECP-H15A. (A) *L. donovani* promastigotes were incubated for 4 h with 4 μM ECP or ECP-H15A and fractionated in a high-sensitivity Experion RNAChip. (B) Densitometric ratio of the different rRNA bands between Ss and the two Ls species. Percentages were referred to the value of the whole densitogram.

To evaluate the contribution of catalytic activity to the antiparasite action, we explored the effect of ECP on cell RNA content. The RNA pattern from promastigotes incubated with either ECP or ECP-H15A was compared with that of control parasites to ascertain whether the RNase was active inside the parasite (Figure 4). The typical trypanosomatid rRNA pattern^28^ of three major, distinctive bands as rRNA large subunit (Ls) breaks into 28 α and 28 β, was notably altered for parasites treated with ECP, in contrast to the unaltered pattern of ECP-H15A-treated promastigotes.

### *L. donovani* Promastigote Accumulates Intracellularly Both ECP and ECP-H15A

**Figure 5:**
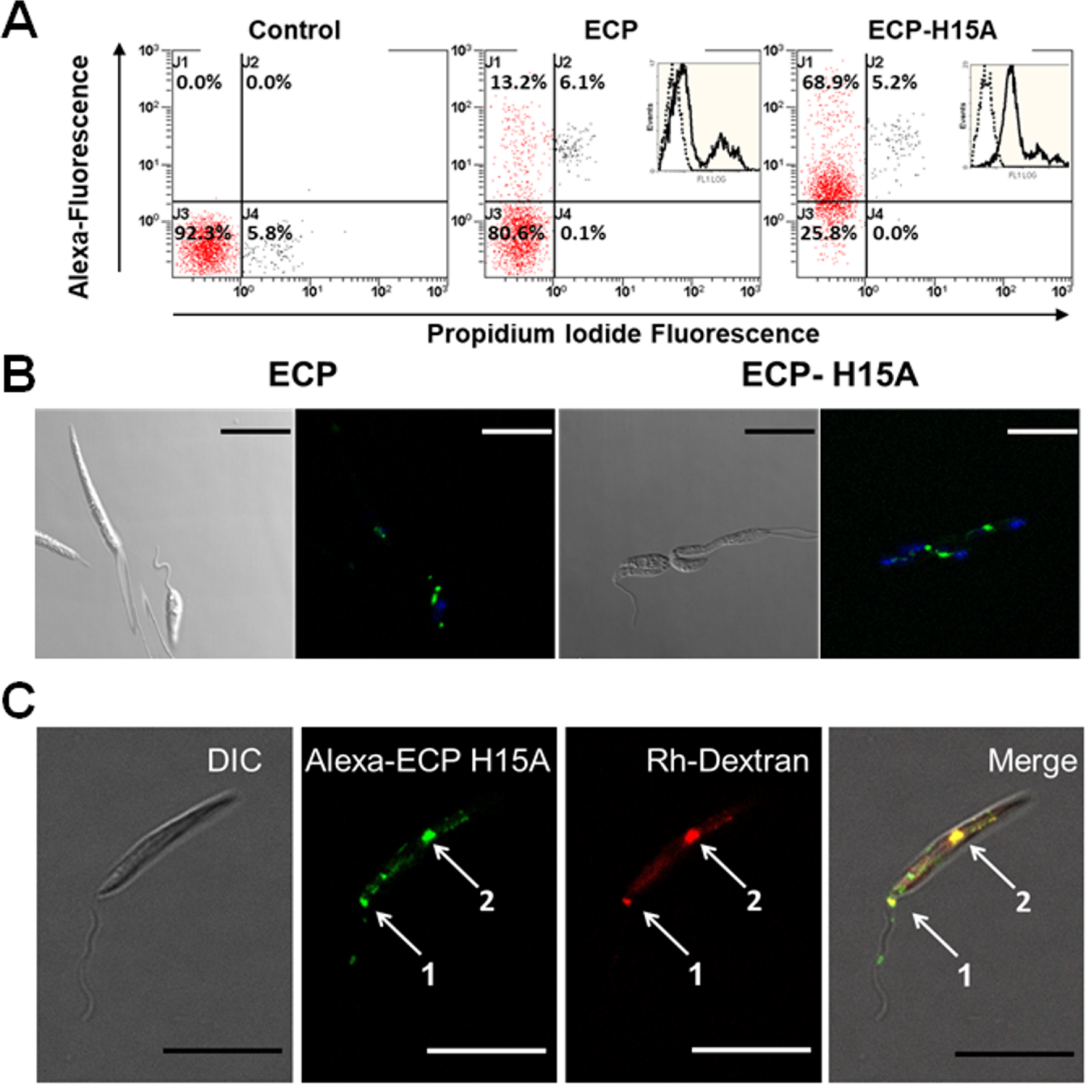
Accumulation and distribution pattern of Alexa 488-labeled ECP or ECP-H15A in *L. donovani* promastigotes. Parasites were incubated with 4 μM ECP or ECP-H15A for 4 h in HBSS-Glc. (A) Protein incorporation measured by cytofluorometry; prior to analysis, propidium iodide was added to detect membrane permeabilization. Insets show the fluorescence distribution of viable parasites (red population), defined by propidium iodide exclusion. (B) Alexa 488-labeled visualization of incorporated proteins (green fluorescence) by confocal microscopy. Parasites were incubated with DAPI 20 min prior to observation to visualize nucleus and kinetoplast, as described in the Methods section. (C) Incorporation of ECP-H15A into *L. donovani* promastigotes. Parasites were incubated with 10 μM Alexa 488-labeled ECP-H15A in the presence of Rhodamine B-dextran (∼70 kDa) (10 mg/mL) for 6 h. Confocal microscopy was carried out with living promastigotes in the presence of Cygel immobilization medium. Pearson correlation coefficient was 0.782 for spot 1, accounting for the flagellar pocket, and of 0.835 for spot 2, likely a large endocytic vacuole. Fluorescent settings: Alexa 488 (λ_exc_ = 488 nm; λ_em_ = 540 nm); DAPI (λ_exc_ = 364 nm; λ_em_ = 454 nm); rhodamine B-dextran (λ_exc_ = 570 nm; λ_em_ = 590 nm). Samples were prepared by duplicate, and experiments were repeated at least twice. Magnification bar = 5 μm. Abbreviations: DIC, differential interference contrast microscopy; Rh-dextran, rhodamine B-dextran.

To further assess whether the leishmanicidal effect was due to RNase activity, and not to a preferential uptake into the parasites for a specific ECP form, we investigated ECP-H15A and ECP internalization by *L. donovani* promastigotes. When Alexa 488-labeled ECP or ECP-H15A were incubated, the accumulation of inactive ECP-H15A was observed, according to cytofluorometry measurements (Figure 5A). We surmise from this that RNA degradation upon uptake of wild-type ECP may alter endocytosis for ECP, but not for ECP-H15A.

A spotted distribution for both fluorescent proteins was observed (Figure 5B). Altogether, this distribution pattern of a spotted accumulation of the two structurally similar ECP variants suggests entry through endocytosis. This was further supported by the high colocalization of rhodamine B-dextran, a typical fluid-phase marker for endocytosis, with labeled ECP-H15A (Figure 5C). ECP-H15A was selected over ECP due to its lower toxicity on parasites and higher uptake.

**Figure 6:**
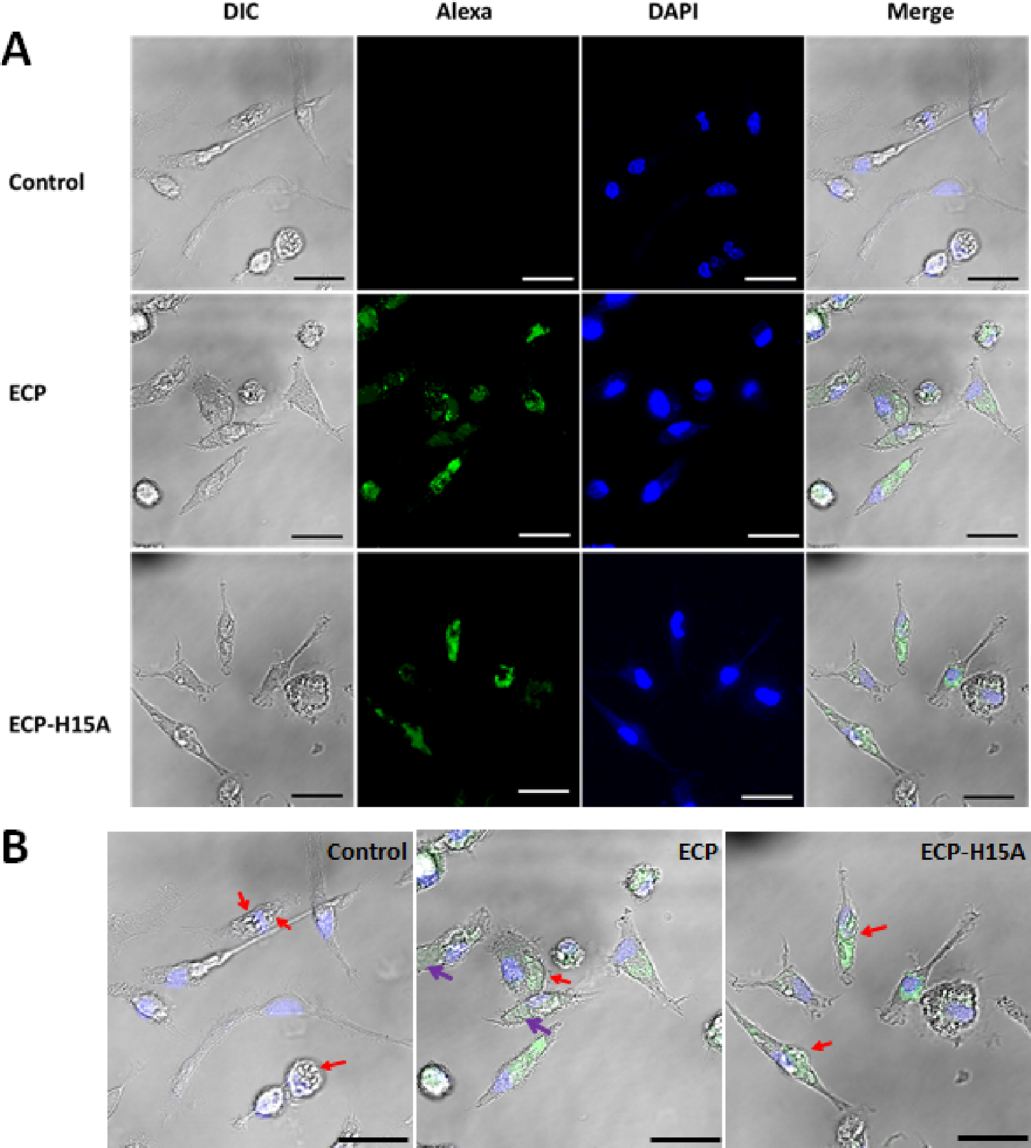
Subcellular distribution of Alexa 488-labeled ECP and ECP-H15A in BALB/c peritoneal macrophages infected with *L. pifanoi* amastigotes. (A) Infected macrophages were incubated with 4 μM labeled ECP or ECP-H15A overnight and observed by confocal microscopy. Fluorescent settings: Alexa 488 (green) (λ_exc_ = 488 nm; λ_em_ = 540 nm); DAPI (blue) (λ_exc_ = 364 nm; λ_em_ = 454 nm). Bar = 25 μm. (B) Magnification of the merge images from (A) to highlight the parasitophorus vacuoles of the three macrophage samples. Red arrows point to parasitophorus vacuoles containing parasites. Purple arrows highlight empty vacuoles, often containing cell debris from killed parasites.

Fluorescence microscopy illustrated the internalization of both ECP and ECP-H15A within infected macrophages. Interestingly, abundance of empty vacuoles was only observed upon addition of wild-type, but not mutant ECP (Figure 6 and Supporting Figure 1).

On the other hand, both versions of the protein differed in their effectiveness to reduce parasite number. The intracellular amastigote ratio decreased from 1.6 ± 0.47 amastigote/macrophage in control parasites to 0.54 ± 0.34 (*p* < 0.01) in macrophages incubated with ECP. For those macrophages incubated with ECP-H15A, this ratio only decreased to 1.21 ± 0.17 (*p* < 0.05).

## Discussion

Antimicrobial proteins and peptides (AMPs) are among the most efficient resources of innate immunity to deter pathogen invasion.^29^ Their broad range of susceptible organisms, along with small size and multitask capabilities (angiogenesis, vascular permeability, priming of antigen-specific response) in addition to primarily microbicidal ones, epitomizes economy of means in biology.

An appealing characteristic of AMPs such as lysozyme^30^ or lactoferrin^31^ is the ability to exert microbicidal activity by more than one mechanism. ECP also exemplifies this duality; thus, microbicidal and cytotoxic properties have been generally ascribed to basic character and membrane-disrupting properties,^32^ not strictly related to RNase activity, while neurotoxicity, induction of cutaneous lesions, or antiviral and antifungal activities are closely associated with RNase action (reviewed in ref (13)). In this context, ECP-H15A is an ideal model to assess the relative contributions of membrane perturbation and RNase mechanisms^14^ since the mutation preserves the former but eliminates the latter.

In the present work, ECP was not fully innocuous to *Leishmania* membrane integrity, as it induced the entry of vital dyes under an all-or-none pattern, similar to its effect on bacteria.^15^ Our results for confocal microscopy on *Leishmania* show that, under the assayed experimental conditions, permeabilization is not caused by massive and stable ECP accumulation at the parasite surface. This may result from local formation of amyloid aggregates on the surface of *Leishmania* cells,^15^ locally perturbing the membrane and favoring endocytosis and further dissemination into the cytoplasm. An alternative explanation could be clustering of acidic phospholipids from the external leaflet of the plasma membrane around inserted ECP molecules. This process would be driven by the high positive charge of ECP, with ensuing alteration of lipid microdomains and faulty phospholipid packing at their boundaries, eventually leading to partial membrane permeabilization in the absence of canonical pore formation.^33^ In any event, RNase activity appears to be required for ECP to exert full leishmanicidal activity. The native protein showed much higher activity than ECP-H15A and, more convincingly, the RNA pattern of parasites exposed to native ECP evidenced notable degradation, while for those incubated with ECP-H15A, no significant alteration was found.

In theory, the intracellular access of ECP into *Leishmania* could be achieved by either direct translocation across the plasma membrane of the parasite, or by endocytosis. ECP is highly cationic and rich in Arg residues, two features common to cell-penetrating peptides (CPPs) that translocate across membranes. Indeed, a CPP comprising ECP residues 32–41 has shown translocation behavior.^34^ Here, we demonstrated that ECP uptake kinetics and its intracellular punctuated distribution, together with its colocalization with rhodamine B-dextran, even in living but immobilized parasites, supports endocytosis as the preferential pathway for ECP uptake, and rules out massive translocation across the plasma membrane. The two bright fluorescence areas spotted within the parasite account for the flagellar pocket, the sole entry zone for endocytosis in trypanosomatids, and the other by an endosome or a lysosome belonging also to the endocytic pathway.

Endocytosis requires a membrane receptor. For *Leishmania*, one might surmise that strongly anionic components of the promastigote such as lipidophosphoglycan (LPG) and proteophosphoglycan (PPG) may act as ECP receptors, not unlike mammalian cells, where anionic polysaccharides of the extracellular matrix interact with ECP.^35^ A potential role for LPG or PPGs as ECP anchor on *Leishmania* would also be consistent with the known involvement of these components in the binding of other AMPs to the parasite prior to its membrane destabilization.^21,36^ This process might also explain our finding of lower susceptibility to ECP in amastigotes, a stage where LPG and PPG expression is considerably reduced.^37^

Once ECP is routed within the endocytic pathway of *Leishmania*, it will gain access to the cytoplasm. Two important facts support *Leishmania* as a highly sensitive target to foreign RNases. First, the pool and variety of putative RNA substrates should be much larger than inferred from the proteome, since the strong post-transcriptional control of gene expression in trypanosomatids^38^ accounts for a constitutive mRNA transcription for most of the proteins; in addition, the whole set of mitochondrial tRNAs are nuclearly encoded.^39^ Second, ECP activity inside *Leishmania* should furthermore be rather independent of control mechanisms on endogenous RNases. Thus, for the RNase inhibitor protein, a ubiquitous intracellular control for RNases in mammalian cells,^40^ genome mining in *Leishmania* finds the highest match in an unclassified protein (XP_001470396) with rather low (25%) similarity, despite a shared leucine-rich repeat motif.

Together with ECP, we have also investigated how peptides known to mimic ECP activity on bacteria^22^ behave against *Leishmania*. The largest analogue, ECP(1–45), reproducing the entire *N*-terminal domain, was 2–3 times less active than native ECP on both promastigotes and in amastigotes; in addition, contrary to ECP, the initial damage was partially recovered at longer exposure times. Smaller-size analogues behaved similarly or worse (Table 1), with the exception of ECP(8–45), with a leishmanicidal profile not unlike that of native ECP.

Although eosinophils phagocytize and kill *Leishmania* promastigotes and amastigotes in vitro,^10^ if ECP is to play a role in the resolution of *Leishmania* infection, it must be active on intracellular parasites. In the present work, when murine peritoneal macrophages infected with *L. pifanoi* were challenged with ECP, the parasite burden was significantly reduced and macrophages with ECP accumulation into the parasitophorus vacuoles in close contact with intracellular amastigotes were spotted. Macrophage viability will be ensured from ECP leakage into the cytoplasm by the endogenous RNase inhibitor (RI) ubiquitously present in the cytosol of vertebrate somatic cells.^40^ Interestingly, recent work demonstrated how ECP can efficiently translocate into macrophages and eradicate the intracellular dwelling mycobacteria through an autophagy pathway.^16^

Macrophages are devoid of granules to store AMPs; however, they may complement their own AMP repertoire by the acquisition of foreign AMPs.^41^ In this respect, ECP can be acquired from eosinophils by macrophages, as it was localized inside vacuoles of alveolar or dermal macrophages from chronic eosinophilic pneumonia^42^ or atopic dermatitis.^43^ In addition, the effectiveness of eosinophil effectors on *Leishmania* was supported by the phagocytosis and further destruction of *L. donovani* promastigotes by eosinophils *in vitro*.^10^

Eosinophil recruitment and degranulation is observed during infections with diverse parasites. Nonetheless, the role of eosinophils in the course of leishmaniasis is still controversial. These cells were traditionally associated with a Th2-driven polarization of the immune response, taken in general as favorable for the progression of *Leishmania* infection. In murine cutaneous leishmaniasis caused by *Leishmania major*, eosinophils preserve the M2 phenotype of the neighboring infected macrophages, permissive for *Leishmania* proliferation, even under the heavy proinflammatory environment of the lesion.^8^ This notion is challenged by other works, however. Recruitment of eosinophils in *Leishmania amazonensis* lesions occurs at later stages of infection, concurrent with parasite clearance.^9^*L. amazonensis* infection in IL-5 transgenic mice, in which eosinophils account for half of the total peripheral lymphocytes, showed an improved infection resolution relative to the parental mice.^11^ Among eosinophil granule proteins, ECP is one of the main ones stored in the secondary granules.^12^

Herein we have disclosed the role of ECP as a feasible leishmanicidal agent to account for the protective role of eosinophils on *Leishmania* infection.^9^ Also, we have shown that ECP leishmanicidal activity can be extended to amastigotes, the pathological form of the parasite, and have thus proposed a dual leishmanicidal mechanism for ECP. Unlike bacterial targets, ECP kills *Leishmania* mostly by its RNase activity, with membrane destabilization playing a secondary role. Altogether, our results provide a proof of concept for the use of RNases from various biological sources as leishmanicidal agents; some are already in clinical trials as antitumoral and anti-HIV agents.^44^ In this context, our laboratory is engineering ECP chimeric variants with enhanced catalytic activity and reduced toxicity.^45^ Along this line, homologous RNases have been converted into effective toxins by simply promoting their intracellular uptake, e.g., through arginine grafting or polyarginine fusion.^46^ Another appealing alternative is the design of ECP variants based on the known natural polymorphisms of the protein; such as the ECP434(G > C)/ ECP-R97T, devoid of cytotoxicity, but with preserved RNase activity. Although the loss of an exposed cationic patch may cripple slightly its uptake, it can no doubt serve as a lead to explore the use of ECP in clinical settings.^47^ In addition, the fact that *Leishmania* is an obligate intravacuolar parasite exclusive for macrophages enables RNase vehiculation into nanoparticles as a way to target ECP into the infection niche at nontoxic doses.^48^

## Conclusions

Our results confirm the versatility of ECP as a vital component of innate immunity. Despite the relatively low RNase activity of ECP, this work evidenced that leishmanicidal activity relies mostly on such enzymatic activity, rather than on an irreversible cell membrane disruption, essential for its bactericidal activity and emulated by its *N*-terminal peptides. In this sense, *Leishmania* is an appealing target for RNases routed into the intracellular space. The constitutive transcription for most of the *Leishmania* genes and the absence of a highly effective cytoplasmic RNase inhibitor, similar to that described for mammalian cells, make *Leishmania* highly vulnerable to RNase attack, even for intracellular parasites. Thus, our results shed light on a possible leishmanicidal mechanism associated with eosinophils, whose role in the *Leishmania* lesion has not been yet fully unveiled. In addition, the ECP as a feasible leishmanicidal agent is a proof of concept of protein-based therapies addressing intracellular targets, paving the way toward the future implementation of minimally engineered host RNases with no toxicity and enhanced RNase activity.

## Methods

### Purification and Characterization of ECP and the Enzymatically Inactive Variant ECP-H15A

A human ECP synthetic gene was cloned into the pET11c expression vector to be further expressed in the *Escherichia coli* BL21(DE3) strain (Novagen, Madison, WI), as previously described.^16^ Briefly, protein was refolded from inclusion bodies and purified by cation exchange (Resource S and Mono S columns, GE Healthcare Europe, Freiburg, Germany) and reverse phase (Vydac C4, Waters, Saint-Quentin, France) chromatographies. The ECP-H15A variant was constructed using the Quick Change site-directed mutagenesis kit (Stratagene, La Jolla, CA). The construct was confirmed by DNA sequencing and the purified protein was analyzed by MALDI-TOF MS and *N*-terminal sequencing.

For fluorescent studies, the proteins were labeled using the Alexa Fluor 488 protein labeling kit (Thermo Fisher, Alcobendas, Spain), according to manufacturer instructions. The labeled enzyme was next desalted through a PD10 column (GE Healthcare, Madrid, Spain) using PBS as an equilibrating and eluting buffer and the extent of reaction was evaluated according to ref (16).

To measure the enzymatic activity of ECP and ECP-H15A, the degradation of oligocytidylic acid was monitored as the variation in the 286 nm absorption in a Cary Eclipse spectrophotometer (Agilent Technologies), as previously described.^14^ Assay conditions were 1 μM protein, 84 μM oligocytidylic acid in 0.2 M NaAcO, pH 5.0, and 3 min incubation at 25 °C.

Membrane leakage activity was assessed by ANTS/DPX (8-aminonaphthalene-1,3,6-trisulfonic acid disodium salt/*p*-xylenebispyridinium bromide).^14^ Large unilamellar vesicles of dioleoyl-phosphatidylcholine:dioleoyl-phosphatidylglycerol (3:2 molar ratio), containing 12.5 mM ANTS plus 45 mM DPX in 20 mM NaCl, 10 mM Tris/HCl, pH 7.5, were diluted to 30 μM and incubated at 25 °C with the proteins for 45 min. Leakage was monitored as the increase in fluorescence (λ_exc_ = 386 nm; λ_em_ = 535 nm).

### Peptides

Peptides ECP(1–45), ECP(8–45), ECP(24–45), and ECP(6–17)-Ahx-(23–36) derived from the *N*-terminal domain of ECP (sequences in Table 1), with native Cys residues replaced by Ser to prevent disulfide formation, have been described.^22^ They were purified to >95% homogeneity by HPLC and satisfactorily characterized by MALDI-TOF MS.

### Parasite Strains

*L. donovani* promastigotes (MHOM/SD/00/1S-2D strain, Prof S. Croft London School of Tropical Medicine and Hygiene) and 3-Luc strain,^26^ and *L. pifanoi* axenic amastigotes strain (MHOM/VE/60/Ltrod, Prof. Diane McMahon-Pratt, Yale University) were grown at 26 or 32 °C for promastigotes or axenic amastigotes, respectively, according to standard procedures.^49^

### Leishmanicidal Activity

Parasites were harvested at the late exponential growth phase, washed twice in Hanks’ buffer supplemented with 10 mM D-glucose (HBSS-Glc) at 4 °C, and then resuspended at 2 × 10^7^ cells/mL in the same buffer. These conditions were maintained for all of the experiments until otherwise stated. Leishmanicidal activity was determined by MTT (3-(4,5-dimethylthiazol-2-yl)-2,5-diphenyltetrazolium bromide) reduction, as described.^49^ Briefly, parasite suspension was aliquoted into a 96-microwell plate (120 μL/well) and incubated with ECP or peptides for 4 h at 27 or 32 °C for promastigotes and amastigotes, respectively. Afterward, a 20 μL aliquot from each well was transferred into a new 96-microwell plate containing 180 μL of growth medium and incubated for 72 h at 26 °C, or 32 °C for 96 h for promastigotes or amastigotes, respectively. To the remaining 100 μL of parasite suspension in each well, MTT (0.5 mg/mL, final concentration) was added immediately after the 4 h incubation, and the resulting formazan was solubilized with 2.5% SDS and read at 595 nm. Samples were assayed in triplicate, and experiments were repeated at least twice. IC_50_ and LC_50_ were calculated using Sigma Plot v. 11 statistical software.

### Variation of Free Cytoplasmic -ATP Levels

Changes in the level of free cytoplasmic ATP in living parasites caused by ECP or its peptides were assessed by a luminescence method using *L. donovani* promastigotes of the 3-Luc strain, expressing a cytoplasmic form of *Photinus pyralis* luciferase.^26^ Briefly, promastigotes were dispensed in HBSS-Glc into a black 96-microwell plate, and DMNEP-luciferin (d-luciferin (1-(4,5-dimethoxy-2-nitrophenyl)) ethyl ester), a free-membrane permeable caged substrate for luciferase, was added (final values: 100 μL/well, 2 × 10^7^ promastigotes/mL, 25 μM DMNEP-luciferin). When luminescence reached a plateau, the respective protein or peptide was added (*t* = 0, luminescence = 100%) and luminescence variation was monitored in a BMG Polarstar Galaxy microwell reader (POLARstar Omega, Öffenburg, Germany) for 30 min and expressed as luminescence percentage at *t* = 0.

### Membrane Permeabilization and Depolarization in *L. donovani* Promastigotes

Depolarization was measured by the increase in fluorescence caused by the insertion of the anionic dye bisoxonol. Promastigotes were resuspended in HBSS-Glc at standard conditions; bisoxonol (0.2 μM, final concentration) and reagents were added at the respective concentration, and readout monitored in a BMG Polarstar Galaxy microwell reader (λ_exc_ = 540 nm; λ_em_ = 580 nm). Full depolarization was achieved by the addition of the membrane-active peptide CA(1–8)M(1–18) at 3 μM.^26^ Entrance of the vital dye SYTOX green (Invitrogen) was assessed under the above standard conditions, with the addition of 1 μM probe in HBSS-Glc medium. After the addition of ECP or peptides, the increase in fluorescence (λ_exc_ = 504 nm; λ_em_ = 524 nm) was monitored. Results were expressed as a percentage relative to cells fully permeabilized with 0.1% Triton X-100.^49^

### Electron Microscopy

Promastigotes were incubated with ECP or ECP(8–45) at 4 μM for 4 h, collected by centrifugation, washed twice with 1 mL of PBS, fixed in 5% (w/v) glutaraldehyde in the same medium, and included with 2.5% (w/v) OsO_4_ for 1 h. Next, the cells were gradually dehydrated in ethanol (30, 50, 70, 90, and 100% (v/v); 30 min each), included with propylene oxide (1 h), embedded in Epon 812 resin, and observed in a Jeol-1230 electron microscope.^36^

### Confocal Microscopy

*L. donovani* promastigotes at 2 × 10^7^ cells/mL were incubated with ECP-Alexa at 4 μM for 4 or 12 h in Hank′s buffer or complete media, respectively. For infected macrophages, only the second condition was used. After incubation, the cells were washed with PBS and labeled with DAPI (5 μg/mL) 10 min before observation in a Leica TCS-SP2 ABOS confocal laser scanning microscope, without fixation. Colocalization experiments were carried out by incubation of promastigotes with 10 μM ECP-H15A Alexa plus 10 mg/mL rhodamine B isothiocyanate dextran (∼70 kDa) for 6 h. Pearson correlation coefficient was obtained with ImageJ software (Version 1.53r).

### Flow Cytometry

*L. donovani* promastigotes were incubated for 4 h at 26 °C with 4 μM ECP-Alexa or ECPH15A-Alexa, then centrifuged at 4 °C, washed twice with 1.5 mL of ice-cold HBSS–Glc, resuspended in the same buffer at 1 × 10^6^ cells/mL, and maintained in ice. Propidium iodide (PI) was added at 5 μg/mL final concentration 2 min before measurements. Samples were analyzed in an FC500 Coulter cytofluorometer (fluorescence settings: PI (λ_exc_ = 488 nm; λ_em_ = 665 nm) and Alexa Fluor −488 (λ_exc_ = 488 nm; λ_em_ = 540 nm)).

### RNA Degradation

An *L. donovani* promastigote suspension (500 μL, 2 × 10^7^ cells/mL) was incubated with 4 μM ECP or ECP-H15A in HBSS-Glc for 4 h. After incubation, the cells were sedimented and resuspended in 1 mL of TRIzol (Invitrogen). Isolation was done according to manufacturer instructions. Samples were analyzed using an Experion high-sensitivity RNAChip (Bio-Rad, Madrid, Spain).

### *L. pifanoi* Infection of Murine Peritoneal Macrophages

BALB/c mice macrophages were elicited by intraperitoneal injection of 1 mL of 4% thioglycollate (Difco Labs, Detroit, MI) followed by peritoneal lavage with PBS 3 days afterward. Animal procedures were carried out according to protocols approved by the Animal Welfare Commission of the CSIC and authorized by the Autonomic Community of Madrid (permission reference: PROEX 070/18). The cells were washed in RPMI-1640 media supplemented with 10% heat-inactivated fetal calf serum and seeded into LabTek culture chamber incubation slides (Thermo Scientific) at 5 × 10^4^ cells per chamber. Next day, adherent macrophages were incubated with *L. pifanoi* amastigotes (4:1 parasite:macrophage ratio) for 4 h. The cells were repeatedly washed until full removal of the nonphagocytized parasites. Infection was allowed to progress for 48 h, followed by treatment with 4 μM ECP-Alexa for 12 h. The lethal effect of the peptide was then assessed by confocal microscopy as the number of parasites per 100 macrophages, averaging from three different fields.

## Supporting Information Available

Confocal microscopy of murine peritoneal macrophages infected with *L. pifanoi* amastigotes incubated with ECP-Alexa (Supporting Figure 1) (PDF)

## Abbreviations

AMP: antimicrobial peptide
CPP: cell-penetrating peptide
ECP: eosinophil cationic protein
EDN: eosinophil-derived neurotoxin
IC_50_: half-maximal inhibitory concentration
HC_50_: half-maximal hemolytic concentration
LC_50_: half-maximal lethal concentration
LPG: lipophosphoglycan
MBP-1: major basic protein-1
PPG: proteophosphoglycan;
Rh-dextran: Rhodamine B-dextran
RI: RNase inhibitor protein
