# Supplementary material for: Essential Role of Enzymatic Activity in the Leishmanicidal Mechanism of the Eosinophil Cationic Protein (RNase 3)
Source: ACS Infect Dis. 2022 Jun 22;8(7):1207–17. doi: 10.1021/acsinfecdis.1c00537 (PMC9274760; doi:10.1021/acsinfecdis.1c00537)
Supplement: Supplementary file 1 — id1c00537_si_001.pdf [file id1c00537_si_001.pdf]

## SUPPORTING INFORMATION

# Essential role of the enzymatic activity for the leishmanicidal mechanism of the eosinophil cationic protein (RNase 3)

**Authors**: María Ángeles Abengózar[a&]; María Fernández-Reyes[a&]; Vivian A. Salazar [b,c], Marc Torrent[b], Beatriz G. de la Torre[d#]; David Andreu[d] *; Ester Boix[b] *; and Luis Rivas[a]*.

[a]Consejo Superior de Investigaciones Científicas. (C.S.I.C.). Centro de Investigaciones Biológicas Margarita Salas. Department of Structural and Chemical Biology. Ramiro de Maeztu 9, 28040, Madrid, Spain.

[b] Department of Biochemistry and Molecular Biology, Faculty of Biosciences, Universitat Autònoma de Barcelona, 08193 Cerdanyola del Vallès, Spain

[c].- Universidad de los Andes, Department of Biomedical Engineering. Cra. 1E No. 19a-40. Bogota, CO 111711. Colombia.

[d] Department of Medicine and Life Sciences, Universitat Pompeu Fabra, Barcelona Biomedical Research Park, Dr. Aiguader 88, 08003 Barcelona, Spain

*Email: luis.rivas@csic.es

Phone: (+34)-911098034

*Email: ester.boix@uab.cat

*Email: david.andreu@upf.edu.

[&] M.A.A and M.F-R contributed equally to the work

2 pages, 1 Figure.

INDEX

Page

**Figure S1**.- Confocal microscopy of murine peritoneal macrophages ............ 2
infected with *L. pifanoi* amastigotes incubated with ECP-Alexa

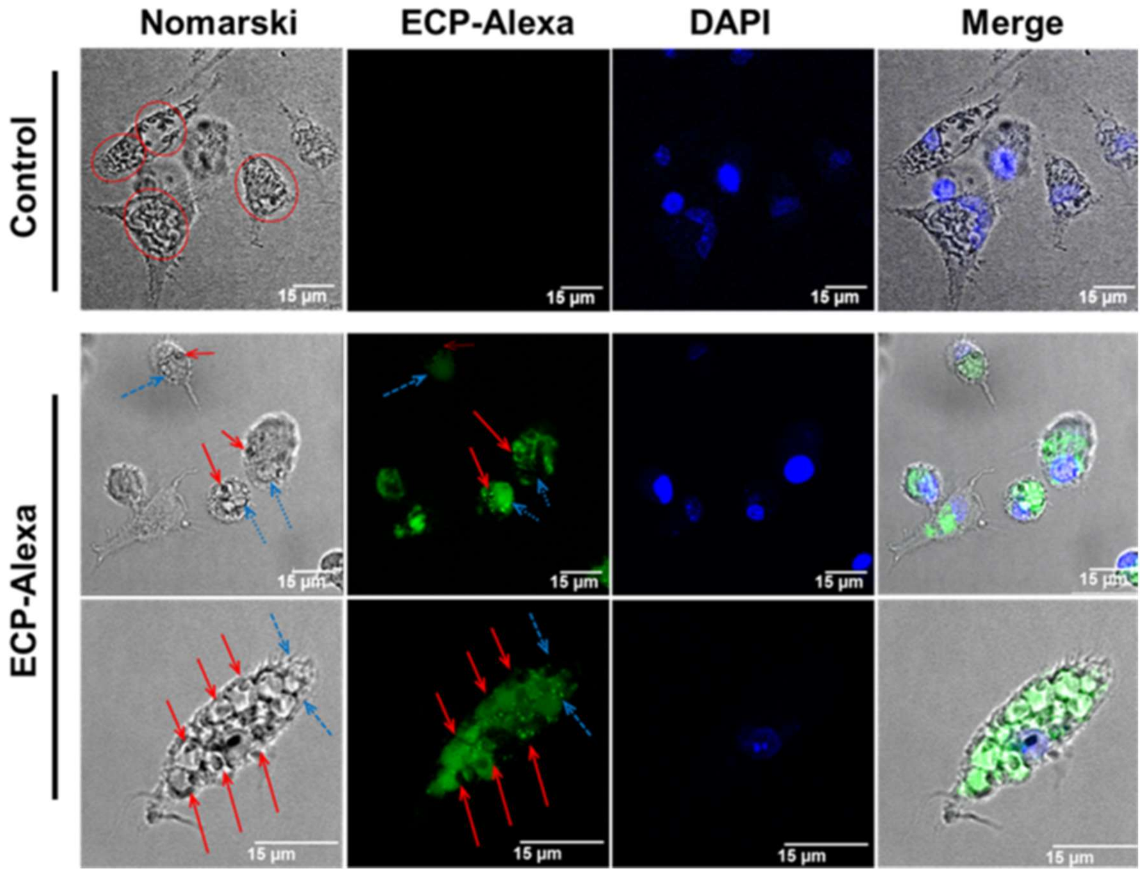


**Figure S1.- Confocal microscopy of murine peritoneal macrophages infected with L. pifanoi amastigotes incubated with ECP-Alexa**. Murine peritoneal macrophages were infected with *L. pifanoi* axenic amastigotes as described in Material and Methods. Once the infection was established, macrophages were treated with 4 µM ECP labelled with Alexa-fluor-488 (Alexa–ECP) for 12 h in complete medium, or remained untreated (control). Red arrows point out to vacuoles with parasites, whereas blue ones do to empty ones. Red circles signal macrophage areas rich in living amastigotes. Fluorescent settings: ECP-Alexa (λexc= 488 nm; λem= 540 nm). DAPI (λexc= 364 nm; λem= 454 nm).
